# Arts participation and mental health: evidence from the Chinese general social survey

**DOI:** 10.3389/fpsyg.2026.1933119

**Published:** 2026-09-11

**Authors:** Chuanjun Liu, Jing Wu

**Affiliations:** School of Fine Arts and Calligraphy, Sichuan Normal University, Chengdu, China

**Keywords:** art participation, mental health, physical health, social connectedness, subjective well-being

## Abstract

Against the backdrop of growing public concern regarding mental health, examining the relationship between arts participation and individual psychological well-being is important for understanding the potential health-related value of sociocultural activities. Drawing on large-scale microdata from the Chinese General Social Survey (CGSS), this study employs ordinary least squares (OLS) regression models to investigate the association between arts participation and individual mental health, while further examining potential mediating pathways and group heterogeneity. The results indicate that, first, arts participation is significantly associated with better mental health, with individuals reporting higher levels of arts participation exhibiting lower frequencies of depressive and dispirited feelings. Second, mediation analyses suggest that social connectedness, subjective well-being, and physical health may constitute important pathways linking arts participation to mental health. Third, heterogeneity analyses reveal marked differences across educational groups, with the positive association between arts participation and mental health being more pronounced among individuals with lower levels of educational attainment. Overall, the findings suggest that arts participation is closely associated with more favorable psychological outcomes and may hold potential value as a sociocultural resource for mental health promotion.

## 1 Introduction

With the accelerating pace of social life and ongoing changes in demographic structures and social environments, mental health has become an increasingly important public health concern (Riedel-Heller et al., 2023). Psychological problems such as anxiety and depression not only impair individuals’ quality of life (Hachuła et al., 2023) but also have broader implications for family well-being and social development (Wang et al., 2023). Consequently, identifying sociocultural resources embedded in everyday life that may contribute to better mental health has attracted growing attention across the fields of public health and the social sciences. Within this context, arts participation has received increasing scholarly interest because it integrates aesthetic experience, emotional expression, and social interaction, all of which may be relevant to psychological well-being.

Existing research has documented a close association between arts participation and individual psychological well-being (Wang et al., 2020). Both arts appreciation and creative engagement may provide opportunities for emotional expression and psychological relaxation, while also fostering positive affect, self-efficacy, and a sense of meaning in life (Zarobe and Bungay, 2017; Ishiguro et al., 2023; Jensen et al., 2023). Among individuals experiencing psychological distress, participation in creative arts has likewise been associated with more favorable mental health outcomes and higher levels of subjective well-being (Caddy et al., 2012). In addition, arts-related activities often involve varying degrees of social interaction, thereby creating opportunities for interpersonal communication and strengthening social connectedness and perceived social support (Cases-Cunillera et al., 2025). Previous studies have also suggested that arts participation may be positively associated with physical health and subjective well-being (Jensen et al., 2023; Fan et al., 2024). Collectively, these findings provide an important empirical foundation for understanding the relationship between arts participation and mental health.

Nevertheless, several gaps remain in the existing literature. First, much of the available evidence is based on specific populations, relatively small samples, or arts-based intervention programs. Large-scale empirical evidence concerning the relationship between arts participation and mental health in the general population remains comparatively limited, particularly in the Chinese context. Second, previous studies have primarily focused on whether arts participation is associated with mental health, whereas the social, psychological, and physical health pathways that may underlie this relationship have received less systematic quantitative examination. Third, access to cultural resources, leisure opportunities, and arts participation varies across social groups. Accordingly, whether the association between arts participation and mental health differs systematically across population subgroups remains an important question requiring further investigation.

To address these gaps, this study draws on data from the Chinese General Social Survey (CGSS) to examine the relationship between arts participation and individual mental health. It further investigates three potential mediating pathways—social connectedness, subjective well-being, and physical health—and explores whether the observed association varies across groups with different levels of educational attainment. By focusing on the Chinese social context, this study aims to provide large-scale micro-level evidence on the relationship between arts participation and mental health, while also offering a more nuanced understanding of its potential correlational pathways and population heterogeneity.

## 2 Theoretical analysis and research hypotheses

### 2.1 Art participation and mental health

Arts participation constitutes a form of cultural engagement that integrates aesthetic experience, emotional expression, and social practice, and may therefore be closely associated with individual mental health. From a theoretical perspective, Maslow’s hierarchy of needs conceptualizes aesthetic needs as an important component of higher-order human needs, suggesting an intrinsic connection between aesthetic experience, self-actualization, and positive psychological functioning. Participation in artistic activities, such as attending concerts, visiting exhibitions, or engaging in creative practice, may provide individuals with opportunities to temporarily disengage from the pressures of everyday life and to experience psychological relaxation and emotional regulation through aesthetic immersion.

At the same time, artistic activities often provide a meaningful channel for emotional expression. During the appreciation or creation of artworks, individuals may develop an emotional resonance with artistic content, thereby facilitating emotional expression, psychological relaxation, and self-reflection, all of which may be associated with lower levels of negative affect, including anxiety and depressive feelings (Wang et al., 2020). From a broader sociocultural perspective, arts participation may also enrich individuals’ cultural and spiritual lives by providing positive life experiences and reinforcing a sense of meaning and purpose. Taken together, these theoretical considerations suggest that higher levels of arts participation may be associated with more favorable mental health outcomes.

Based on the above reasoning, the following hypothesis is proposed:

*Hypothesis 1*: Arts participation is significantly associated with individual mental health, such that higher levels of arts participation are associated with lower levels of depressive and dispirited feelings.

### 2.2 The mechanism and path by which art participation affects mental health

The association between arts participation and mental health is unlikely to operate through a single pathway. Accordingly, this study further examines three potential mediating pathways—social connectedness, subjective well-being, and physical health—that may help explain the relationship between arts participation and mental health.

#### 2.2.1 Social connection

Social connectedness refers to stable and meaningful interpersonal ties between individuals and others and represents an important social resource closely associated with mental health. According to social support theory, high-quality social relationships can provide emotional, informational, and instrumental support, thereby helping individuals cope with everyday stressors and adverse life events.

Artistic activities, particularly collective forms of participation such as choral singing, community theater, and calligraphy or painting groups, inherently involve varying degrees of social interaction. Such activities provide participants with opportunities to communicate and interact around shared interests, meet others with similar preferences, exchange artistic experiences, and establish new social ties (Perkins et al., 2021; Cases-Cunillera et al., 2025). Even receptive forms of arts participation, such as attending performances or visiting exhibitions, may involve shared experiences and subsequent interactions with family members or friends, thereby creating additional opportunities for social engagement.

The interpersonal interactions and social ties associated with arts participation may strengthen individuals’ sense of belonging, emotional support, and perceived security. Stronger social connectedness, in turn, is generally associated with lower levels of loneliness and more favorable psychological states. From a theoretical perspective, social connectedness may therefore represent an important pathway linking arts participation to mental health.

Based on the above reasoning, the following hypothesis is proposed:

*Hypothesis 2a*: Social connectedness may constitute an important mediating pathway in the association between arts participation and individual mental health.

#### 2.2.2 Subjective well-being

Subjective well-being refers to individuals’ overall cognitive and affective evaluations of their lives according to their own standards and is commonly reflected in life satisfaction, positive affective experiences, and the regulation of negative affect. Unlike mental health as operationalized in this study, which focuses primarily on negative psychological states such as depressive and dispirited feelings, subjective well-being more directly captures individuals’ positive evaluations of life and the psychological resources available to them. Subjective well-being may therefore represent an important psychological pathway linking arts participation to mental health.

Arts participation may be positively associated with subjective well-being through several processes. On the one hand, arts appreciation often elicits positive emotional experiences, including pleasure, tranquility, and emotional resonance, which may allow individuals to achieve a degree of psychological detachment from everyday stress and negative affect. On the other hand, artistic creation and active arts engagement may foster absorption, a sense of accomplishment, and self-efficacy, while also enabling emotional expression and meaning-making. These psychological experiences may be associated with greater life satisfaction and higher levels of subjective well-being (Lee et al., 2021; Fan et al., 2024).

In turn, higher subjective well-being generally reflects greater access to positive emotions and psychological resources. Individuals who evaluate their lives more positively may be better equipped to regulate their emotions and recover psychologically when confronted with stressors and adverse life events, and may consequently report lower levels of depressive and dispirited feelings.

From this theoretical perspective, arts participation may be associated with higher subjective well-being, while greater subjective well-being may itself be associated with more favorable mental health outcomes. Subjective well-being may therefore serve as an important psychological pathway connecting arts participation with mental health.

Based on the above reasoning, the following hypothesis is proposed:

*Hypothesis 2b (H2b)*: Subjective well-being may constitute an important mediating pathway in the association between arts participation and individual mental health.

#### 2.2.3 Physical health condition

Mental and physical health are closely intertwined. Poor physical health is frequently accompanied by greater psychological distress and more negative emotional experiences, whereas relatively favorable physical health may provide an important foundation for maintaining psychological stability. Consistent with perspectives emphasizing mind–body interdependence, physiological and psychological states are closely and reciprocally related (Węziak-Białowolska and Białowolski, 2016; Liu et al., 2025).

Some forms of arts participation also entail varying degrees of physical engagement. Activities such as dancing, singing, and playing musical instruments typically involve bodily movement or physical exertion to differing extents (Jensen et al., 2023). In addition, the pleasurable and relaxing experiences associated with arts participation may be related to more favorable perceptions of one’s own physical condition. From this perspective, individuals who participate more frequently in artistic activities may also report better perceived physical health.

Better physical health, in turn, is generally associated with more positive psychological functioning and lower levels of depressive and dispirited feelings. Physical health may therefore constitute another potentially important pathway through which arts participation is associated with mental health.

Based on the above reasoning, the following hypothesis is proposed:

*Hypothesis 2c (H2c)*: Physical health may constitute an important mediating pathway in the association between arts participation and individual mental health.

## 3 Research design

### 3.1 Sample selection

The empirical analysis draws upon data from the Chinese General Social Survey (CGSS), a comprehensive, nationwide survey administered by the China National Survey Data and Analysis Center at Renmin University of China. To ensure national representativeness, the survey employs a rigorous, multi-stage stratified sampling design based on map-assisted address selection—a methodology widely recognized as the gold standard in large-scale socio-economic inquiries within China. The dataset encompasses 29 provinces, municipalities, and autonomous regions. This study harmonizes six waves of the survey, spanning 2012, 2013, 2015, 2017, 2018, and 2021. To guarantee the validity of the regression estimates, observations with missing values for key variables—including the core explanatory variables, dependent variables, mediators, and controls—were excluded. The final analytical sample consists of 59,201 observations.

### 3.2 Variable selection

#### 3.2.1 Dependent variable

The dependent variable in this study is individual mental health (MH), which is operationalized by measuring the frequency of negative emotions—specifically, feelings of depression and distress. This variable is derived from the following survey question: “During the past 4 weeks, how often have you felt depressed or distressed?” Responses were recorded on a five-point Likert scale, with options ranging from “never” to “always,” coded as 1 through 5, respectively. It is important to note that this measure is reverse-coded, such that higher scores indicate a greater frequency of depressive symptoms and, consequently, poorer mental health.

#### 3.2.2 Core explanatory variables

The core explanatory variable in this study is art participation (AP), which is derived from the following survey question: “In the past year, how often did you participate in cultural activities, such as attending concerts, performances, or exhibitions?” Responses were assessed using a five-point Likert scale, with the options “never,” “once a year or less,” “several times a month,” “several times a week,” and “every day” assigned values of 1 through 5, respectively. Higher scores indicate a greater frequency of art participation.

#### 3.2.3 Mediating variables

The variable of social connection (SC) is employed to capture the frequency and intensity of an individual’s interactions within their social network. This measure is derived from the following survey question: “In the past year, how often did you spend time with friends during your leisure time?” Responses were recorded on a five-point Likert scale, with the options “never,” “once a year or less,” “several times a month,” “several times a week,” and “every day” assigned values of 1 through 5, respectively. Higher values on this measure indicate a greater frequency of social interaction with friends, reflecting a stronger degree of social connection.

The variable of subjective well-being (SW) is designed to capture an individual’s overall evaluation and satisfaction with their quality of life. This measure is derived from the core survey question: “Overall, do you consider yourself a happy person?” Responses were assessed using a five-point Likert scale, with the options “very unhappy,” “somewhat unhappy,” “neutral,” “somewhat happy,” and “very happy” assigned values of 1 through 5, respectively. Higher values on this measure indicate a greater level of subjective well-being.

The variable of physical health status (PHS) is employed to capture an individual’s subjective perception and evaluation of their current health condition. This measure is derived from the core survey question: “How would you rate your current physical health?” Responses were assessed using a five-point Likert scale, with the options “very unhealthy,” “somewhat unhealthy,” “fair,” “somewhat healthy,” and “very healthy” assigned values of 1 through 5, respectively. Higher values on this measure indicate a more favorable self-perceived physical health status.

#### 3.2.4 Control variables

To account for potential confounding factors, this study draws on the existing literature to select a set of individual-level characteristics that may influence mental health as control variables. Specifically, these control variables are defined and justified as follows.

Gender (GEN) is coded as 1 for male respondents and 0 for female respondents, consistent with well-established evidence that gender heterogeneity systematically shapes individual artistic engagement preferences and mental health status (Sher et al., 2025; Oktariani, 2026).

Age (AGE) is constructed as the discrepancy between the survey year and each respondent’s birth year, which captures generational disparities in artistic participation tendencies and psychological well-being; distinct cohort discrepancies in depression susceptibility and cultural consumption patterns are consistently observed across age groups.

Religious belief (RB) is dichotomized as 1 for individuals with religious affiliations and 0 for those without, as religious orientation fundamentally reshapes personal psychological adjustment mechanisms and cultural participation predispositions. Religious adherents exhibit heterogeneous emotion regulation strategies and higher propensities to engage in religion-related artistic activities, justifying its inclusion as a valid control (Zhou, 2022; Hou et al., 2023).

Residence registration (RR) takes a value of 1 for agricultural household registration and 0 for non-agricultural household registration. Constrained by China’s urban–rural dual institutional structure, individuals with different registration statuses exhibit substantial disparities in public cultural resource accessibility and artistic participation opportunities, accompanied by divergent mental health formation mechanisms across urban and rural subsamples (He et al., 2024).

Marital status (MS) is coded as 1 for married individuals and 0 for unmarried individuals. Serving as a pivotal source of social and emotional support, marital status generates stable discrepancies in affective resource endowments and cultural participation behaviors, thereby representing a standard covariate in relevant psychological and sociological research.

Political affiliation (PA) is ordinally coded as 1 for the general public, 2 for Communist Youth League members, 3 for democratic party members, and 4 for Communist Party members. Political identity profoundly reflects individual social involvement intensity and organizational belongingness, wherein varying levels of social capital accumulation and collective participation frequency jointly generate confounding effects on both artistic engagement behaviors and subjective mental health perceptions (Choi et al., 2023).

Income (INC) is measured by the natural logarithm of respondents’ total annual income in the preceding year. As a fundamental material prerequisite for cultural consumption and artistic participation, income directly determines individual economic affordability for cultural activities; meanwhile, its robust correlation with mental health renders income a core socioeconomic control variable for mitigating omitted variable bias.

### 3.3 Model specification

This study employs ordinary least squares (OLS) regression models to empirically examine the association between arts participation and mental health. The baseline model is specified as follows:


$${\mathrm{MH}}_{\mathrm{it}}=\alpha_{0}+\alpha_{1}AP_{\mathrm{it}}+\text{βControl}+\mu_{i}+v_{i}+\varepsilon_{\mathrm{it}}$$ (1)

In Equations (1), where the *i* and *t* denote province and year, respectively; *MH_it_* represents the dependent variable of mental health; *AP_it_*is the core explanatory variable of art participation; Control denotes the set of control variables; *α* and *β* are the corresponding regression coefficients; μ_i_ captures province fixed effects, while υ_t_ accounts for year fixed effects; and ε_it_ is the stochastic error term.

### 3.4 Descriptive statistical analysis

Table 1 presents the descriptive statistics for the main variables. The final analytical sample comprises 59,201 observations. The mean value of the mental health variable (MH) is 2.1353, which is below the midpoint of the five-point scale, suggesting that respondents generally report relatively favorable psychological states and, on average, experience depressive or dispirited feelings only infrequently. However, the standard deviation of 0.9805 indicates substantial variation in reported psychological states across individuals.

**Table 1:** Descriptive statistics of variables.

Variable	Obs	Mean	Std. Dev.	Min	Max
MH	59,201	2.1353	0.9805	1.0000	5.0000
AP	59,201	1.4219	0.7508	1.0000	5.0000
SC	59,201	2.3499	0.9878	1.0000	5.0000
SW	59,201	3.8574	0.8327	1.0000	5.0000
PHS	59,201	3.5581	1.0885	1.0000	5.0000
GEN	59,201	0.4874	0.4998	0.0000	1.0000
AGE	59,201	50.6548	16.6129	17.0000	118.0000
RB	59,201	0.1104	0.3133	0.0000	1.0000
RR	59,201	0.5473	0.4978	0.0000	1.0000
MS	59,201	0.8684	0.3380	0.0000	1.0000
PA	59,201	1.3933	0.9651	1.0000	4.0000
INC	59,201	8.3920	3.6214	0.0000	16.1181

The mean value of arts participation (AP), the core explanatory variable, is 1.4219, which is considerably below the midpoint of the scale. This suggests that the overall level of arts participation in the sample is relatively low, with the average respondent reporting a frequency close to “never.” Its standard deviation of 0.7508 likewise indicates considerable heterogeneity in the frequency of arts participation across individuals.

Taken together, the descriptive statistics suggest that respondents generally report relatively favorable psychological states but relatively low levels of arts participation. These distributional characteristics provide an empirical basis for the subsequent examination of the association between arts participation and mental health.

## 4 Empirical analysis

### 4.1 Baseline regression analysis

Table 2 reports the results of the baseline regression analysis. Province fixed effects and year fixed effects are included in all model specifications. Specifically, Model (1) presents the estimates without individual-level control variables, while Model (2) retains the same specification but clusters standard errors at the provincial level. Models (3) and (4) further incorporate individual-level covariates into Models (1) and (2), respectively.

**Table 2:** Results of baseline regression analysis.

Variable	(1)	(2)	(3)	(4)
AP	−0.0612***	−0.0612***	−0.0198***	−0.0198**
	(0.0054)	(0.0116)	(0.0055)	(0.0097)
GEN			−0.1034***	−0.1034***
			(0.0082)	(0.0108)
AGE			0.0071***	0.0071***
			(0.0003)	(0.0009)
RB			0.0739***	0.0739**
			(0.0133)	(0.0278)
RR			0.1140***	0.1140***
			(0.0092)	(0.0133)
MS			−0.1292***	−0.1292***
			(0.0129)	(0.0155)
PA			−0.0496***	−0.0496***
			(0.0042)	(0.0046)
INC			−0.0153***	−0.0153***
			(0.0012)	(0.0016)
Constant	2.2223***	2.2223***	2.0953***	2.0953***
	(0.0086)	(0.0165)	(0.0214)	(0.0398)
Province FE	Yes	Yes	Yes	Yes
Year FE	Yes	Yes	Yes	Yes
*N*	59,201	59,201	59,201	59,201
*R* ^2^	0.043	0.043	0.070	0.070

Standard errors in parentheses, *p < 0.1, **p < 0.05, ***p < 0.01.

Across all specifications, the estimated coefficient on arts participation, the core explanatory variable, is negative and statistically significant. Given that higher values of the dependent variable indicate more frequent depressive and dispirited feelings, these results suggest that, after accounting for individual characteristics as well as province and year fixed effects, higher levels of arts participation are significantly associated with lower frequencies of depressive and dispirited feelings.

Overall, the findings indicate a stable statistical association between arts participation and individual mental health. Individuals who participate more frequently in artistic activities tend to report more favorable psychological states. These results are consistent with the theoretical expectation formulated in Hypothesis 1.

### 4.2 Robustness analysis

#### 4.2.1 Variable replacement and model switching

In the baseline regression, the dependent variable—frequency of depressive symptoms—was measured using a five-point Likert scale, with responses ranging from “never” to “always” coded as 1 through 5, respectively. This variable is reverse-coded, such that higher scores indicate a greater frequency of depressive symptoms, corresponding to lower levels of mental health.

To assess the sensitivity of the estimation results to the measurement approach, we conduct the following robustness checks. First, we transform the continuous variable into a binary variable, categorizing “never” and “rarely” as “mentally healthy” (coded as 0), and “sometimes,” “frequently,” and “always” as “mentally unhealthy” (coded as 1), and re-estimate the model using ordinary least squares (OLS). The results are presented in Model (1) of Table 3. Second, in light of the ordinal discrete nature of the dependent variable, we re-estimate the model using a Logit specification, with the results shown in Model (2) of Table 3.

**Table 3:** Robustness test.

Variable	(1)	(2)	(3)	(4)	(5)	(6)
	Replace the explained variable	Logit	Exclude special age groups	Exclude samples from municipalities	Nearest neighbor matching	Caliper matching
AP	−0.0152***	−0.0769***	−0.0134**	−0.0228**	−0.0138***	−0.0163***
	(0.0046)	(0.0241)	(0.0052)	(0.0107)	(0.0049)	(0.0048)
Constant	0.3355***	−0.7949***	0.3130***	2.0933***	0.3447***	0.3178***
	(0.0184)	(0.1065)	(0.0222)	(0.0416)	(0.0206)	(0.0184)
Control	Yes	Yes	Yes	Yes	Yes	Yes
Province FE	Yes	Yes	Yes	Yes	Yes	Yes
Year FE	Yes	Yes	Yes	Yes	Yes	Yes
*N*	59,201	59,201	47,697	48,313	25,735	40,814
*R* ^2^	0.052		0.049	0.061	0.040	0.050

Standard errors in parentheses, *p < 0.1, **p < 0.05, *** *p* < 0.01.

The robustness analyses yield coefficients on arts participation that are consistent in direction with those obtained from the baseline regressions and remain statistically significant at the 1% level. This indicates that the main findings are robust to alternative measurements of the dependent variable and alternative model specifications.

#### 4.2.2 Elimination of special samples

To account for potential systematic differences in the factors influencing mental health among certain subpopulations, we conduct robustness checks by excluding specific sample groups. First, we exclude individuals under the age of 18 and those over 65, as their mental health status is more susceptible to physiological development and aging-related factors. Second, we remove observations from the four 直辖市的 municipalities—Beijing, Shanghai, Tianjin, and Chongqing—where public cultural resources are disproportionately concentrated, leading to systematic differences in the accessibility of art participation compared to other regions.

Regression results following the exclusion of these specific subsamples are presented in Models (3) and (4) of Table 3. The findings show that the sign and statistical significance of the coefficient for art participation remain largely consistent with those of the baseline regression, suggesting that the main findings are not driven by the inclusion of these particular subsamples.

#### 4.2.3 Preference score matching method

To mitigate endogeneity concerns arising from sample selection bias, this study employs propensity score matching (PSM) to preprocess the sample. Specifically, propensity scores are first estimated based on individual characteristics. A 1:1 nearest-neighbor matching procedure is then applied to identify matched samples from the control group (non-participants) for the treatment group (art participants), and the average treatment effect on the treated (ATT)—that is, the average net effect of art participation on mental health—is subsequently estimated. As a robustness check, caliper matching with a caliper radius of 0.01 is also conducted.

The nearest-neighbor matching results indicate that the total sample size prior to matching was 59,201 observations, which reduced to an effective sample of 25,735 observations after matching. The balance diagnostics reveal that following matching, the standardized bias for all covariates fell below 5%, and the t-tests failed to reject the null hypothesis of no significant differences in covariates between the treatment and control groups. These findings suggest that systematic differences between the two groups were effectively eliminated, confirming the adequacy of the matching procedure. Regression results based on the matched sample are presented in Model (5) of Table 3.

The caliper matching results show that the effective sample size after matching comprises 40,814 observations. The balance diagnostics similarly indicate that the standardized bias for the majority of covariates falls below 5%, demonstrating satisfactory matching performance. Regression results based on the matched sample are presented in Model (6) of Table 3.

The results obtained using both matching approaches further show that the coefficient on arts participation remains negative and statistically significant at the 5% level. Thus, after reducing observed differences between participants and non-participants through matching, arts participation remains significantly associated with more favorable mental health outcomes. These findings provide additional evidence for the robustness of the baseline results.

### 4.3 Mechanism analysis

The baseline regression results reveal a significant statistical association between arts participation and individual mental health. Building on this finding, the present study employs mediation models to examine whether social connectedness, subjective well-being, and physical health may constitute potential pathways linking arts participation to mental health.

In the first stage, we examine the association between arts participation and each potential mediator. The model is specified as follows:


$${\mathrm{MH}}_{\mathrm{it}}=\alpha_{0}+\alpha_{1}AP_{\mathrm{it}}+\text{βControl}+\mu_{i}+v_{i}+\varepsilon_{\mathrm{it}}$$ (2)

In the second stage, we examine the association between each mediating variable and mental health. The model is specified as follows:


$${\mathrm{MH}}_{\mathrm{it}}=\alpha_{0}+\alpha_{1}M_{\mathrm{it}}+\text{βControl}+\mu_{i}+v_{i}+\varepsilon_{\mathrm{it}}$$ (3)

Finally, arts participation and the mediating variable are simultaneously included in the regression model to examine their respective associations with mental health and to assess changes in the coefficient of arts participation after the mediator is introduced. The model is specified as follows:


$${\mathrm{MH}}_{\mathrm{it}}=\alpha_{0}+\alpha_{1}AP_{\mathrm{it}}+\alpha_{2}AP_{\mathrm{it}}+\text{βControl}+\mu_{i}+v_{t}+\varepsilon_{\mathrm{it}}$$ (4)

In Equations (2–4), *M_it_* denotes the mediating variable, which in this study refers to social connectedness, subjective well-being, and physical health. The results of the mediation analyses are reported in Table 4.

**Table 4:** Results of mechanism analysis.

Variable	(1)	(2)	(3)	(4)	(5)	(6)	(7)	(8)	(9)
	SC	MH	MH	SW	MH	MH	PHS	MH	MH
AP	0.2208***		−0.0081	0.0578***		−0.0005	0.0680***		0.0054
	(0.0084)		(0.0095)	(0.0070)		(0.0090)	(0.0088)		(0.0085)
SC		−0.0542***	−0.0531***						
		(0.0069)	(0.0067)						
SW					−0.3348***	−0.3348***			
					(0.0059)	(0.0060)			
PHS								−0.3715***	−0.3717***
								(0.0055)	(0.0055)
Constant	2.7089***	2.2277***	2.2392***	3.5545***	3.2845***	3.2853***	4.3941***	3.7377***	3.7287***
	(0.0323)	(0.0379)	(0.0381)	(0.0241)	(0.0540)	(0.0517)	(0.0439)	(0.0365)	(0.0360)
Control	Yes	Yes	Yes	Yes	Yes	Yes	Yes	Yes	Yes
Province FE	Yes	Yes	Yes	Yes	Yes	Yes	Yes	Yes	Yes
Year FE	Yes	Yes	Yes	Yes	Yes	Yes	Yes	Yes	Yes
N	59,201	59,201	59,201	59,201	59,201	59,201	59,201	59,201	59,201
*R* ^2^	0.158	0.072	0.072	0.044	0.147	0.147	0.203	0.205	0.205

Standard errors in parentheses, *p < 0.1, **p < 0.05, ***p < 0.01.

#### 4.3.1 Social connections

This subsection examines whether social connectedness may constitute a potential mediating pathway between arts participation and mental health.

As shown in Model (1), the first-stage regression yields a coefficient of 0.2208 for arts participation, which is statistically significant at the 1% level. This finding indicates a significant positive association between arts participation and social connectedness, such that individuals with higher levels of arts participation tend to report stronger social connectedness.

The second-stage regression results, presented in Model (2), show that the coefficient on social connectedness is −0.0542 and statistically significant at the 1% level. Given that higher values of the dependent variable indicate more frequent depressive and dispirited feelings and, consequently, less favorable mental health, this result suggests that stronger social connectedness is significantly associated with lower levels of depressive and dispirited feelings. In other words, individuals with greater social connectedness tend to exhibit more favorable psychological states.

Model (3) simultaneously includes arts participation and social connectedness. The coefficient on social connectedness remains negative and statistically significant at the 1% level, with an estimated value of −0.0531. Meanwhile, the coefficient on arts participation decreases from −0.0198 in the baseline regression to −0.0081 and is no longer statistically significant.

Taken together, these changes in the estimated coefficients suggest that, in statistical terms, social connectedness may represent an important pathway linking arts participation to mental health. Higher levels of arts participation are significantly associated with stronger social connectedness, which in turn is associated with lower levels of depressive and dispirited feelings. These findings are consistent with the theoretical pathway proposed in Hypothesis 2a.

#### 4.3.2 Subjective well-being

This subsection further examines whether subjective well-being may constitute a potential mediating pathway between arts participation and mental health.

Model (4) assesses the association between arts participation and subjective well-being. The estimated coefficient on arts participation is 0.0578 and statistically significant at the 1% level, indicating that individuals with higher levels of arts participation tend to report greater subjective well-being. This finding is consistent with the preceding theoretical discussion, which suggests that arts participation may be accompanied by positive emotional experiences, psychological engagement, life satisfaction, and a stronger sense of meaning.

Model (5) further examines the relationship between subjective well-being and mental health. The coefficient on subjective well-being is −0.3348 and statistically significant at the 1% level, indicating that individuals with higher levels of subjective well-being tend to report lower levels of depressive and dispirited feelings. This result is consistent with the theoretical expectation that greater subjective well-being is generally associated with more positive psychological resources and more favorable emotional states.

When arts participation and subjective well-being are simultaneously included in Model (6), the coefficient on subjective well-being remains −0.3348 and statistically significant at the 1% level, whereas the coefficient on arts participation decreases from −0.0198 in the baseline regression to −0.0005 and is no longer statistically significant.

Taken together, the pattern of coefficients reveals a significant positive association between arts participation and subjective well-being and a significant negative association between subjective well-being and depressive and dispirited feelings. Moreover, the estimated coefficient on arts participation decreases substantially after subjective well-being is introduced into the model. These results suggest that, in statistical terms, subjective well-being may constitute an important mediating pathway linking arts participation to mental health.

In other words, individuals who participate more frequently in artistic activities also tend to report higher levels of subjective well-being, while higher subjective well-being is, in turn, associated with lower levels of depressive and dispirited feelings. This empirical pattern is consistent with the theoretical pathway proposed in Hypothesis 2b.

#### 4.3.3 Physical health condition

This subsection further examines whether physical health may constitute a potential mediating pathway between arts participation and mental health.

The regression results indicate a statistically significant association between arts participation and physical health, providing an empirical basis for further examining the potential mediating role of physical health.

Subsequent regression results show that the coefficient on physical health is −0.3715 and statistically significant at the 1% level. Because higher values of the mental health measure correspond to more frequent depressive and dispirited feelings, this finding indicates that individuals reporting better physical health tend to exhibit lower levels of depressive and dispirited feelings. Thus, physical health is significantly associated with the psychological outcome examined in this study.

When arts participation and physical health are simultaneously included in the model, the coefficient on arts participation changes from −0.0198 in the baseline regression to 0.0054 and becomes statistically insignificant, whereas the coefficient on physical health is −0.3717 and remains statistically significant at the 1% level.

Taken together, these findings suggest that, in statistical terms, physical health may constitute a potential mediating pathway between arts participation and mental health. The observed pattern of associations among arts participation, physical health, and depressive and dispirited feelings is broadly consistent with the theoretical expectation underlying Hypothesis 2c. Accordingly, the empirical results provide support for Hypothesis 2c.

### 4.4 Heterogeneity analysis

To examine whether the association between arts participation and mental health varies across educational groups, the sample is divided according to respondents’ highest level of educational attainment into a lower-education group (below junior college) and a higher-education group (junior college or above). Separate regression analyses are then conducted for the two subsamples, with the results reported in Table 5.

**Table 5:** Results of heterogeneity analysis.

Variable	(1)	(2)
	Low educational level	High educational level
AP	−0.0220**	−0.0132
	(0.0096)	(0.0159)
Constant	2.1063***	2.0372***
	(0.0419)	(0.0515)
Control	Yes	Yes
Province FE	Yes	Yes
Year FE	Yes	Yes
*N*	50,578	8,623
*R* ^2^	0.072	0.025

Standard errors in parentheses, *p < 0.1, **p < 0.05, ***p < 0.01.

Among respondents with lower levels of education, the estimated coefficient on arts participation is −0.0220 and statistically significant at the 1% level. By contrast, among respondents with higher levels of education, the coefficient is −0.0132 and does not reach conventional levels of statistical significance. These findings suggest that the negative association between arts participation and depressive and dispirited feelings is more evident among individuals with lower educational attainment. In other words, within this group, higher levels of arts participation are more clearly associated with lower frequencies of depressive and dispirited feelings.

Several explanations may account for this pattern.

First, the substitutability of cultural and leisure resources may differ across educational groups. Individuals with higher educational attainment may have access to a broader range of cultural, leisure, and social activities, including reading, travel, physical exercise, and other forms of social engagement. Because these activities may themselves be associated with more favorable psychological states, the independent association attributable to arts participation may be comparatively limited (Hübner and Pfost, 2025). By contrast, some individuals with lower educational attainment may have access to a narrower range of cultural and leisure opportunities, making the association between arts participation and mental health more salient.

Second, individuals with different educational backgrounds may differ in their modes of arts engagement, motivations for participation, and evaluations of artistic experiences. Those with higher levels of education may be exposed to a wider variety of artistic activities and may also hold more diverse expectations and evaluative standards regarding such experiences. These differences may contribute to variation in the relationship between arts participation and mental health across educational groups.

Third, the nature of cultural and psychological needs, as well as the ways in which such needs are fulfilled, may differ by educational attainment. For some individuals with lower levels of education, arts participation may represent an important avenue for emotional relaxation, affective expression, and social interaction. Accordingly, the association between arts participation and psychological well-being may be more pronounced in this group (Fancourt and Steptoe, 2019).

Overall, the heterogeneity analysis indicates that the relationship between arts participation and mental health is not uniform across educational groups, with a more pronounced association observed among individuals with lower levels of educational attainment.

## 5 Discussion

### 5.1 Theoretical contributions

Drawing on data from the Chinese General Social Survey, this study examines the relationship between arts participation and individual mental health. The findings indicate that, after accounting for individual characteristics as well as province and year fixed effects, higher levels of arts participation are significantly associated with lower levels of depressive and dispirited feelings. This finding is broadly consistent with previous research on the relationship between arts engagement and psychological well-being. Prior studies have shown that arts participation can provide opportunities for emotional expression, self-reflection, and positive experiences and is closely associated with subjective well-being, self-efficacy, and more favorable psychological states (Fan et al., 2024). The present study extends this literature by providing evidence from a large-scale Chinese social survey, suggesting that the positive association between arts participation and mental health is observable not only among specific clinical populations or within arts-based intervention programs but also in the broader general population.

The mediation analyses further suggest that social connectedness, subjective well-being, and physical health may constitute important pathways linking arts participation to mental health. First, artistic activities often involve a degree of social interaction and provide opportunities for communication, collaboration, and shared experiences. Accordingly, individuals with higher levels of arts participation tend to report stronger social connectedness, which is in turn associated with lower levels of depressive and dispirited feelings. This pattern is consistent with prior research showing that arts participation is associated with stronger social ties and greater social support (Perkins et al., 2021; Cases-Cunillera et al., 2025).

Second, arts participation is significantly associated with higher levels of subjective well-being, while greater subjective well-being is itself associated with more favorable psychological states (Lee et al., 2021; Fan et al., 2024). This suggests that positive emotional experiences, life satisfaction, and a sense of meaning may represent important psychological pathways through which arts participation is related to mental health. Finally, physical health also exhibits a potential mediating role, a finding that is consistent with previous research documenting associations between arts participation and physical health (Jensen et al., 2023).

It is important, however, to interpret these mediation findings as theoretically grounded associational pathways rather than as empirically established causal chains. Because the cross-sectional nature of the data does not permit the temporal ordering of arts participation, the mediating variables, and mental health to be determined with certainty, the contribution of the present study lies primarily in identifying statistical relationships that are consistent with theoretical expectations. These findings therefore provide an empirical basis for future research using longitudinal data or more rigorous causal identification strategies to examine these pathways more directly.

The heterogeneity analysis further indicates that the association between arts participation and mental health is more evident among individuals with lower levels of educational attainment. This pattern may reflect differences in access to cultural resources, leisure opportunities, and modes of arts engagement across educational groups. Individuals with higher levels of education often have access to a broader array of leisure and cultural activities, which may reduce the marginal association of arts participation within their overall lifestyle (Hübner and Pfost, 2025). By contrast, for some individuals with lower levels of education, arts participation may constitute a particularly important avenue for emotional regulation, social interaction, and cultural engagement (Fancourt and Steptoe, 2019). Accordingly, the relationship between arts participation and mental health may be more salient among groups with relatively limited cultural and social resources. Nevertheless, this interpretation remains theoretically informed and speculative, as the subgroup regression results themselves do not establish the mechanisms underlying these differences.

From a theoretical perspective, this study makes three principal contributions. First, by situating arts participation at the intersection of the sociology of art and the sociology of health, the study provides large-scale empirical evidence from the Chinese context and extends a literature that has predominantly focused on Western societies and specific population groups. Second, rather than examining only the overall association between arts participation and mental health, the study investigates three potential pathways—social connectedness, subjective well-being, and physical health—thereby providing a more nuanced account of why arts participation may be related to individual psychological states. Third, the analysis of educational heterogeneity shows that the relationship between arts participation and mental health is not evenly distributed across social groups, highlighting the possibility that this association is socially stratified.

The substantive magnitude of the findings should also be interpreted with caution. Although arts participation is statistically significant in most model specifications, both the absolute magnitude of the estimated coefficients and the overall *R^2^* values are relatively small, suggesting that the association between arts participation and mental health is modest in magnitude. Individual mental health is shaped by a wide range of factors, including economic circumstances, family relationships, social support, physical health, major life events, and individual psychological characteristics. Arts participation should therefore be understood as only one potentially relevant sociocultural factor among many.

Accordingly, statistical significance should not be equated with substantial clinical significance. Nor do the present findings imply that arts participation can substitute for professional psychological services or medical treatment. A more appropriate interpretation is that arts participation may function as an everyday sociocultural resource with potential complementary value within broader approaches to mental health promotion.

### 5.2 Limitations and future research directions

Several limitations of this study should be acknowledged.

First, the CGSS adopts a repeated cross-sectional design. Although the present study controls for province fixed effects, year fixed effects, and a range of individual-level characteristics and further conducts several robustness checks, the research design cannot fully address reverse causality or endogeneity arising from unobserved factors. Accordingly, the findings primarily reflect statistical associations between arts participation and psychological states and should not be interpreted as evidence of strict causal effects. Future research could employ longitudinal panel data, quasi-natural experiments, instrumental-variable approaches, or other research designs with stronger causal identification properties to examine the causal relationship more rigorously.

Second, the core variables used in this study are primarily based on respondents’ self-reports and are therefore potentially subject to perceptual biases, recall errors, and social desirability effects. This concern is particularly relevant to depressive and dispirited feelings and physical health, as respondents’ subjective assessments may not fully correspond to their objective psychological or physiological conditions. Future studies could combine survey responses with standardized psychological scales, clinical assessments, physiological indicators, or medical records to improve measurement validity and accuracy.

Third, this study uses respondents’ reported frequency of depressive and dispirited feelings to represent their psychological state. Mental health, however, is a multidimensional construct encompassing emotional states, psychological functioning, social adaptation, and subjective well-being. The frequency of depressive and dispirited feelings primarily captures negative affect or depressive tendencies and cannot fully represent the broader construct of mental health. Accordingly, the conclusions of this study regarding “mental health” should primarily be interpreted as reflecting the relationship between arts participation and negative psychological states such as depressive and dispirited feelings, rather than being generalized to all dimensions of mental health. Future research could employ multidimensional measures incorporating depression, anxiety, positive affect, life satisfaction, and psychological functioning to provide a more comprehensive assessment of mental health.

In addition, the measure of arts participation used in this study primarily captures overall participation frequency and does not distinguish among specific art forms, levels of engagement, or motivations for participation. In practice, different forms of artistic activity—such as music, dance, theater, painting, calligraphy, and exhibition attendance—may differ substantially in the degree of physical activity, social interaction, and emotional experience they entail. Consequently, their relationships with psychological states and their potential mediating pathways may also vary. This distinction may be particularly important for the physical-health pathway, as physically engaging forms of arts participation may differ considerably from more sedentary forms of artistic appreciation. Owing to limitations in the available data, the present study is unable to examine these differences in greater detail. Future research should therefore make use of more fine-grained measures of arts participation to investigate heterogeneity across specific art forms.

## 6 Conclusion

Arts participation represents an important form of sociocultural engagement and is closely associated with individual mental health. Using data from the Chinese General Social Survey (CGSS), this study examines the relationship between arts participation and individual mental health and further explores potential mediating pathways and group heterogeneity.

The findings indicate a significant association between arts participation and mental health, with individuals reporting higher levels of arts participation generally exhibiting lower frequencies of depressive and dispirited feelings. The mediation analyses further suggest that social connectedness, subjective well-being, and physical health may constitute important pathways underlying this association. Specifically, higher levels of arts participation are associated with stronger social connectedness, greater subjective well-being, and better perceived physical health, each of which is in turn significantly associated with lower levels of depressive and dispirited feelings. These findings provide empirical evidence for understanding the social, psychological, and physical-health pathways that may connect arts participation with mental health.

The heterogeneity analysis additionally shows that the association between arts participation and mental health varies across educational groups and is more pronounced among individuals with lower levels of educational attainment. This finding suggests that the relationship between arts participation and mental health may exhibit meaningful population heterogeneity.

Based on these findings, this study proposes the following policy recommendations:

First, strengthen top-level design by integrating art-promoted mental health into public cultural development planning. Given the significant positive effect of art participation on mental health—particularly through the mechanisms of social connection, subjective well-being, and physical health—governments should regard art activities as an important public health intervention. When formulating public cultural service policies, full consideration should be given to their positive role in social psychological construction; coordinated planning is needed to guide and support the popularization and development of various art activities.

Second, optimize resource allocation to reduce group-level disparities in art participation. The finding that art participation exerts a stronger mental health effect among lower-educated groups highlights the need to focus on equity in cultural resource distribution. Governments should increase cultural investment in rural, remote, and low-income areas. By means such as mobile cultural services and the sharing of digital cultural resources, barriers to art participation can be lowered, thereby safeguarding the basic cultural rights of disadvantaged groups and enabling them to benefit equally from art activities.

Third, innovate activity formats to integrate art into community daily life. Communities serve as the fundamental unit of residents’ daily lives and important arenas for fostering social connection. Community-based organizations should be encouraged to organize diverse art activities that respond to residents’ needs, such as public cultural performances, interest-based groups, and art lectures. In this way, art can become a bridge for neighborhood interaction and emotional communication, thereby enhancing residents’ sense of well-being and belonging in their everyday lives and contributing to a positive community mental health ecosystem.

## Data Availability

The datasets supporting the study are publicly available on the CGSS website http://cgss.ruc.edu.cn. Further inquiries can be directed to the corresponding authors.
